# Inactivation Methods for Experimental Nipah Virus Infection

**DOI:** 10.3390/v14051052

**Published:** 2022-05-15

**Authors:** Lina Widerspick, Cecilia Alejandra Vázquez, Linda Niemetz, Michelle Heung, Catherine Olal, András Bencsik, Christoph Henkel, Anneke Pfister, Jesús Emanuel Brunetti, Indre Kucinskaite-Kodze, Philip Lawrence, César Muñoz Fontela, Sandra Diederich, Beatriz Escudero-Pérez

**Affiliations:** 1 WHO Collaborating Centre for Arbovirus and Haemorrhagic Fever Reference and Research, Bernhard Nocht Institute for Tropical Medicine, 20359 Hamburg, Germany; lina.widerspick@bnitm.de (L.W.); linda.niemetz@bnitm.de (L.N.); michelle.heung@bnitm.de (M.H.); olal@bnitm.de (C.O.); bencsik@bnitm.de (A.B.); christoph.henkel@bnitm.de (C.H.); anneke.pfister@t-online.de (A.P.); emanuel.brunetti@bnitm.de (J.E.B.); munoz-fontela@bnitm.de (C.M.F.); 2 German Center for Infection Research (DZIF), Partner Site Hamburg-Luebeck-Borstel-Reims, 38124 Braunschweig, Germany; 3 Instituto de Química Biológica de la Facultad de Ciencias Exactas y Naturales (IQUIBICEN), Consejo Nacional de Investigaciones Científicas y Técnicas-Universidad de Buenos Aires, Ciudad Universitaria, Buenos Aires 1428, Argentina; cvazquez@qb.fcen.uba.ar; 4 Life Science Center, Vilnius University, Sauletekio 7, LT-10257 Vilnius, Lithuania; indre.kodze@bti.vu.lt; 5 Science and Humanities Confluence Research Center (EA 1598), Catholic University of Lyon (UCLy), 69002 Lyon, France; plawrence@univ-catholyon.fr; 6 Institute of Novel and Emerging Infectious Diseases, Friedrich-Loeffler-Institut, 17493 Greifswald-Insel Riems, Germany; sandra.diederich@fli.de

**Keywords:** Nipah virus, inactivation, syncytia, BSL-4, immunofluorescence, RT-qPCR, plaque assay

## Abstract

Nipah virus (NiV) is an emerging zoonotic paramyxovirus that causes severe disease in humans and livestock. Due to its high pathogenicity in humans and the lack of available vaccines and therapeutics, NiV needs to be handled in biosafety level 4 (BSL-4) laboratories. Safe inactivation of samples containing NiV is thus necessary to allow further processing in lower containment areas. To date, there is only limited information available on NiV inactivation methods validated by BSL-4 facilities that can be used as a reference. Here, we compare some of the most common inactivation methods in order to evaluate their efficacy at inactivating NiV in infected cells, supernatants and organs. Thus, several physical and chemical inactivation methods, and combinations thereof, were assessed. Viral replication was monitored for 3 weeks and NiV presence was assessed by RT-qPCR, plaque assay and indirect immunofluorescence. A total of nineteen methods were shown to reduce NiV infectious particles in cells, supernatants and organs to undetectable levels. Therefore, we provide a list of methods for the safe and efficient inactivation of NiV.

## 1. Introduction

Approximately 70% of emerging infectious diseases (EIDs) for humans are zoonoses [1,2], with single-stranded RNA (ssRNA) viruses being a particularly important causative group [3,4]. Amongst them, Nipah virus (NiV) is one of the deadliest pathogens known to affect humans. It can also infect a wide range of wild and domestic animals, which facilitates its transmission to humans. The virus causes severe and highly contagious disease in domestic animals such as pigs, resulting in significant economic losses for farmers [5,6]. In humans, it causes pulmonary or encephalitic-mediated disease, with a case-fatality rate of up to 92% [7,8].

NiV is categorized as a biosafety level 4 (BSL-4) pathogen due to its lethality, its ease of transmissibility between species, and the fact that there are no licensed treatments or vaccines available for either humans or swine. Consequently, NiV is listed in the World Health Organization (WHO) R&D Blueprint list of priority pathogens [9].

Maximum-containment laboratories are, therefore, essential in handling potentially infectious NiV-containing samples for diagnostics and for NiV research. To date, there are very limited published data on how to effectively and safely inactivate NiV-containing samples with different methods in BSL-4 laboratories. For example, it has been shown that ice-cold, absolute methanol can inactivate NiV-infected cells; however, the details of inactivation, such as the inactivation time, were not specified [10]. Recently, two additional chemical inactivation methods, including 4% paraformaldehyde (PFA) for 15 min for cell monolayers and 10% neutral-buffered formalin (NBF) for NiV-infected organs from ferrets, showed complete inactivation of the infectious virus [11]. Other procedures shown to effectively inactivate NiV-infected cells include the heat and ultraviolet (UV) inactivation of sera previously spiked with NiV [12], desiccation, and pH-dependent inactivation [13]. It is also known that, under optimal conditions, NiV can persist for several days in blood, culture media, urine and flesh fruit [13,14]. While it is essential to make sure that viruses are inactivated, it is frequently also necessary to ensure that, during the inactivation process, viral proteins and/or nucleic acid are at least partially preserved for quantification or for research purposes. Most of the inactivation methods tested for NiV so far do not always sufficiently preserve viral proteins or do not include the most common procedures used in laboratories, allowing further processing for diagnostics and research.

In this article, we evaluate the efficacy of two physical inactivation methods, namely UV and heat inactivation, and eleven chemical inactivation methods: PFA, Cytofix/Cytoperm (C/C), acetone, methanol, acetone/methanol, SDS, RLT + ethanol (EtOH), Triton-X 100, Trizol, AVL + EtOH and NBF. We used cell culture supernatants (seven methods), cell cultures (ten methods) and organs (two methods), resulting in a total of nineteen inactivation procedures tested. Therefore, this article provides a list of inactivation procedures to safely handle NiV samples outside BSL-4 containment. 

## 2. Materials and Methods

### 2.1. Cell Culture and Infections

Vero E6 cells (ATCC, Vero C1008, clone E6) were grown in Dulbecco′s modified Eagle′s medium (DMEM; PAN-Biotech, Aidenbach, Germany) supplemented with 100 U/mL Pen/Strep (Gibco; Thermo Fisher Scientific, Paisley, UK) and 5% heat-inactivated fetal bovine serum (FBS; Gibco; Thermo Fisher Scientific, Paisley, UK) at 37 °C and 5% CO_2_. Cells were propagated and seeded for (i) infection to test the inactivation methods and syncytia formation, (ii) propagation of the cells containing the inactivated inoculum or positive control, and (iii) an indirect immunofluorescence (IF) assay. See each specific section for further details.

NiV (Malaysia strain) was kindly provided by Markus Eickmann from the Institute for Virology at the Philipps University Marburg (Marburg, DE). NiV was amplified on Vero E6 cells, where cells were infected at a multiplicity of infection (MOI) of 0.01. At day 3 post-infection (p.i.), the collected medium was centrifugated at 1600 rpm for 5 min to pellet cell debris and supernatant was aliquoted and frozen at −80 °C. NiV titer was determined by plaque assay (PA) under methylcellulose overlay. The virus stock used for all the experiments was lower than 3 passages. All work with infectious NiV was performed under BSL-4 laboratory conditions at the Bernhard Nocht Institute for Tropical Medicine (Hamburg, Germany).

In order to determine a time-course for NiV infection, a total of 5 × 10^4^ cells were plated on glass coverslips and infected at an MOI of 0.01. Cells were fixed at 8, 18, 24 and 48 h p.i. with acetone/methanol (1:1) for 1 h at room temperature (RT). Infected NiV cells were detected by immunofluorescence, as explained in Section 2.3.

In order to produce the cells and the supernatants required to test the inactivation methods, 2 × 10^7^ Vero E6 cells seeded in 175 cm^2^ flasks were infected at an MOI of 0.01 and, after 3 days, cells (fully detached due to the cytopathic effect (CPE)) and supernatant were centrifuged at 1600 rpm for 5 min. Cells and supernatant were kept separate for the following inactivation procedures. Mock infections for the negative control were also performed the same day by adding DMEM only, and were further processed in the same way as infected samples. The resulting infected cells and their negative controls were then used to validate the inactivation methods. A small aliquot of the cells was taken at this first step to verify the infection levels of the samples before inactivation. Supernatants were also kept in several aliquots to determine the initial NiV infection titers and to test their infectious capacity by immunofluorescence. During the propagation phase, supernatants from each inactivation method were kept and cells were carefully washed with PBS solution (PAN-Biotech, Aidenbach, Germany). This PBS wash was then added to the supernatant to avoid losing potentially infected cells. Remaining attached cells were trypsinized and centrifuged to pellet all of the potentially infected cells. Following centrifugation, the cell pellet was resuspended in 5 mL of the corresponding collected supernatant and an inoculation volume of 1 mL was added to fresh Vero E6 cells in a 75 cm^2^ flask containing 1 × 10^6^ cells. After 7 days, the same process of virus propagation was repeated. In total, propagation took place for 21 days following primary inoculation. Additionally, in parallel to the propagation phase, an aliquot of 500 µL of supernatant was added to pre-seeded cells in 24-well plates to analyze the infectious capacity of the inoculum by immunofluorescence. All T75 flasks were extensively monitored for the presence of CPE/syncytia over the course of the propagation phase with light microscopy.

### 2.2. Virus Inactivation

Validation of all the inactivation methods tested was performed in independent triplicates (R1, R2 and R3) with appropriate positive (PC) and negative controls (NC). The replicates (infected cells or supernatant) and NCs (non-infected cells or supernatant) were treated equally during the inactivation processes. In the case of the PC, the inactivating reagents were replaced by PBS. A summary of the nineteen inactivation conditions tested can be found in Table 1.

#### 2.2.1. Inactivation of NiV-Infected Cells

A total of 2 × 10^7^ NiV-infected cells were used per condition (R1-3, PC and NC) during the validation of all inactivation methods involving cells. 

##### 2.2.1.1. Inactivation Methods without Cell Lysis: PFA, Cytofix/Cytoperm (C/C), Acetone, Methanol, Acetone/Methanol

**Figure 1:**
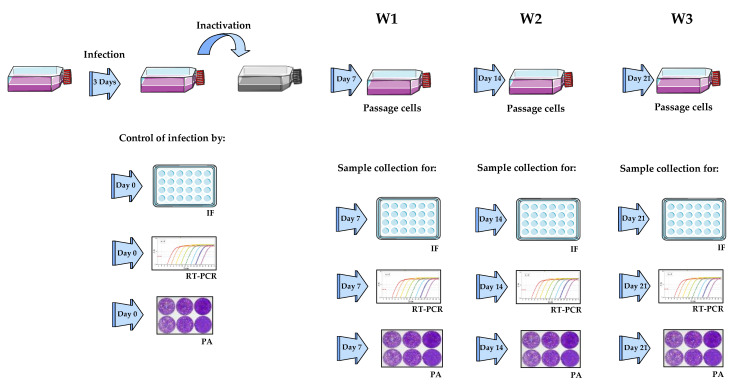
Schematic representation of collection points and analysis techniques during inactivation methods. Vero E6 cells were infected with NiV and 3 days p.i.; samples were collected before inactivation to determine the infection levels. After the inactivation procedures, three time points were established at the first (W1), second (W2) and third (W3) weeks post-inactivation and samples were analyzed by immunofluorescence (IF) to visualize the infectious capacity contained in the samples; RT-qPCR was used to semi-quantify NiV RNA; and the plaque assay (PA) was used to determine the viral titer. Figure created with smart.servier.com.

After centrifugation to remove the supernatant, cells were resuspended with 2 mL of the tested reagents, namely 4% PFA (Sigma-Aldrich, Darmstadt, Germany) for either 30 or 60 min, or 30 min with either C/C (BD Biosciences), acetone (Carl Roth, Karlsruhe, Germany), methanol (Sigma-Aldrich, Darmstadt, Germany) or acetone/methanol at a 1:1 *v*/*v* ratio. After incubation, cells were washed three times with 50 mL of PBS and resuspended in 1 mL of DMEM 5%. This 1 mL of cells was then added to fresh Vero E6 cells for propagation for 3 × 7 days, resulting in a total of 21 days, as described above. In parallel, samples were collected for further analysis by RT-qPCR, immunofluorescence, and plaque assay. See Figure 1 for details.

##### 2.2.1.2. Inactivation Methods with Cell Lysis: SDS, RLT + EtOH, Triton-X 100 and Trizol

After the centrifugation of cells, the supernatant was removed and the cells were inactivated using different lysis methods.

For SDS inactivation, SDS buffer (2× Laemmli Sample Buffer, BioRad, Feldkirchen, Germany) containing 2.1% of SDS and supplemented with 5% 2-mercaptoethanol (Gibco; Thermo Fisher Scientific, Paisley, UK) was used (final concentration of 1× *v*/*v*). Cells pellets were resuspended in 1 mL of SDS buffer and incubated for 10 min at 95 °C.

RLT + EtOH inactivation was performed according to the RNeasy Mini Kit manufacturer’s instructions (Qiagen, Hilden, Germany). Briefly, cells were resuspended in 1.2 mL of RLT buffer and incubated for 10 min at RT. A total of 1.2 mL of 70% EtOH was then added to each sample and the samples were vortexed until complete homogenization. 

For Triton-X 100 inactivation, cells were inactivated with 1 mL of Triton-X 100 (Carl Roth, Karlsruhe, Germany) for 20 min at RT.

For Trizol (Ambion; Thermo Fisher Scientific, Paisley, UK) inactivation, cells were incubated with 2 mL of Trizol reagent at RT and vortexed until they were homogenized.

After incubation with the inactivation reagents, samples were then washed with 200 mL of PBS using Amicon^®^Ultra-15 100K Centrifugal Filter Devices (Millipore, Burlington, MA, USA), as described by Olschewski et al. (2021) [15]. This method has been shown to efficiently remove the inactivating agents while concentrating the sample. Briefly, samples were resuspended in 200 mL of PBS and centrifuged at 3500× *g* for 10 min until the volume was reduced to 1–2 mL. The concentrated samples were then added to freshly prepared uninfected Vero E6 cells that were propagated for 21 days (propagation phase). Simultaneously to the cell propagation phase, samples were collected for further analysis by RT-qPCR, immunofluorescence, and plaque assay. See Figure 1 for details.

#### 2.2.2. Inactivation of NiV-Infected Supernatants: UV Light, SDS, AVL + EtOH, Triton-X 100, Triton-X 100 + Heat and Heat

Validation of the inactivation methods involving supernatants was performed on virus stocks at a final titer of 1 × 10^6^ PFU/mL of NiV in DMEM supplemented with 5% FBS when added to the inactivating reagent.

For UV light inactivation, 5 mL of supernatant was put in a Petri dish and, with the lid open, was exposed to UV light (Sankyo Denki G30T8 Germicidal Lamp, 30 W, AKA 254 nm) inside a Class II laminar hood for 1 h. The distance from the UV lamp was approximately 10 cm.

For SDS inactivation, 500 µL of SDS buffer (described in Section 2.2.1.2) was incubated (1:1) with 500 µL of supernatant for 10 min at 95 °C.

AVL + EtOH inactivation was performed according to the QIAamp Viral RNA Mini kit manufacturer’s instructions. Briefly, 182 µL of supernatant was resuspended in 910 µL of AVL buffer (Qiagen, Hilden, Germany) and incubated for 10 min at RT. Then, 910 µL of absolute EtOH (Carl Roth, Karlsruhe, Germany) was added to each sample and vortexed until it was homogenized. 

For Triton-X 100 inactivation, 500 µL of supernatant was incubated for 20 min at RT with 500 µL of Triton-X 100.

For the Triton-X 100 + heat inactivation method, 500 µL of the virus solution was incubated with 500 µL of Triton-X 100 for 30 min at 56 °C.

For the heat inactivation method, 1 mL of supernatant was incubated for either 30 or 60 min at 56 °C.

After incubation with the inactivation reagents, samples were then washed with 200 mL of PBS using Amicon^®^Ultra-15 100K Centrifugal Filter Devices, as explained in Section 2.2.1.2, propagated for 21 days, and processed further by RT-qPCR, immunofluorescence, and plaque assay. See Figure 1 for details. 

#### 2.2.3. Inactivation of NiV-Infected Organs: Trizol and NBF

Validation of virus inactivation in organs was performed on the lungs of previously infected mice. The virus titer in the lungs ranged from 3 to 5 times 10^7^ PFU/g.

During Trizol inactivation, 0.2 g of lung tissue was added to a tube containing 1 mL of DMEM and Lysing matrix D (MP Biomedicals, Schwerte, Germany) and was homogenized using a FastPrep-24^TM^ 5G tissue lysis homogenizer. Then, 100 µL of homogenate were added to 900 µL of Trizol and were incubated for 20 min at RT. After incubation with the inactivation reagents, samples were washed as explained in Section 2.2.1.2.

For inactivation with 10% neutral-buffered formalin (NBF, ProTaqs), two half lungs were incubated in 2 mL of 10% NBF for 24 h at RT. This volume was enough to cover the whole organ. The other halves were titered to confirm NiV infection. After 24 h, NBF was changed for a fresh solution and left for 24 more hours. Finally, NBF was removed, and the organs were washed twice with 2 mL of PBS and then left in 2 mL of freshly added PBS for 48 h to reduce the presence of formalin in order to avoid toxicity in the propagation phase. Organ samples were then added directly to cell cultures for virus propagation experiments.

The inactivated and washed samples were added to fresh Vero E6 cells that were propagated for 21 days. In parallel to the cell propagation phase, samples were collected for further analysis by RT-qPCR, immunofluorescence, and plaque assay. See Figure 1 for details.

### 2.3. NiV Antibody Production

Antibody-producing hybridoma was developed according to the standard procedure described in [16] by fusing spleen cells of immunized BALB/c mice with mouse myeloma Sp 2/0-Ag14 cells. Briefly, three eight-week-old BALB/c mice were immunized with chimeric antigen (unpublished, kindly provided by prof. Kestutis Sasnauskas): the major capsid protein VP1 of mouse polyomavirus was used as a carrier harboring a 35 aa segment (aa 486–520, accession No. Q9IK92) of NiV N protein. During the immunization process, 50 µg/mouse of recombinant chimeric antigen dissolved in PBS was subcutaneously injected three times every 28 days. For the primary and the second immunization, the antigen was additionally emulsified in complete Freund′s adjuvant (Sigma-Aldrich, St. Louis, MO, USA) and incomplete Freund′s adjuvant (Sigma-Aldrich, St. Louis, MO, USA), respectively.

### 2.4. Immunofluorescence

A total of 5 × 10^4^ Vero E6 cells were seeded in 24-well plates on sterile coverslips (number 1, 12 mm, Assistent). The following day, a 500 µL aliquot of the media contained in the T75 flasks that had been inoculated with the inactivated viral samples for 7 days was added to each well. After 24 h, cells were fixed with 1 mL of acetone/methanol (1:1) solution per well and the 24-well plates were placed in an acetone/methanol container where they were fully covered under high-containment conditions. After one hour, cells were further processed outside the BSL-4. Following fixation, the coverslips were allowed to dry in a chemical hood to remove the organic solvents and were rinsed two times with PBS. Samples were blocked for 30 min at RT with 1% bovine serum albumin in PBS and rinsed once with PBS. Immunolabeling of NiV nucleoprotein N was carried out by incubating samples with the in-house mouse 5F12 primary antibody (1:700 dilution) for 1 h at RT, followed by three washes with PBS. Cells were then incubated for 1 h with Alexa Fluor 594 labelled donkey anti-mouse IgG antibody (3 drops/mL; Invitrogen; Thermo Fisher Scientific, Waltham, USA) or with Alexa Fluor 647 goat anti-mouse IgG secondary antibodies (1:1000 dilution, Thermo Fischer Scientific, Waltham, MA, USA) in the syncytia time-course experiment. Finally, coverslips were washed twice with PBS and once with water and mounted on glass slides with Glycerol Mounting Media with DAPI and DABCO (Abcam, Berlin, Germany). Cells were imaged using a Zeiss Axio Scope Imager M1 microscope. Each sample was scanned to look for the presence of infected cells and, afterwards, three random fields were imaged. Image processing was carried out using Fiji [17].

### 2.5. RT-qPCR

RNA extractions were performed using the QIAamp Viral RNA Mini Kit (Qiagen, Hilden, Germany) according to the manufacturer’s instructions. Briefly, 140 µL of supernatant of each sample was added to 560 µL of AVL and samples were frozen at −80 °C. For extraction, samples were thawed and 560 µL of absolute EtOH (Carl Roth) was added and vortexed to yield a homogenized solution. The extraction protocol was followed according to the manufacturer’s instructions. Finally, columns were incubated with 60 µL of nuclease-free water (Qiagen, Hilden, Germany) for 1 min at RT, centrifuged at 8000 rpm for 1 min, and aliquoted in two volumes of 30 µL.

In order to quantify the RNA contained in the samples, real-time, reverse-transcription polymerase chain reaction (RT-qPCR) targeting the NiV N gene was performed, as described by Feldman et al. [18]. Reaction products obtained from the amplification were semi-quantified based on the standard curves generated using NiV RNA isolated from a virus stock with a known titer.

### 2.6. Plaque Assay

Confluent monolayers of Vero E6 cells were infected with serial 10-fold dilutions of the inactivated samples (3 replicates, a negative control and a positive control). After 1 h of incubation at 37 °C, the inoculums were removed and 1 mL of overlay containing 1% methylcellulose (Sigma-Aldrich, Darmstadt, Germany) medium was added. Plates were incubated for three days at 37 °C and 5% CO_2_. The overlay was then removed and cells were fixed with a 4.5% formaldehyde solution (SAV Liquid Production GmbH). After washing with water to remove the remaining formaldehyde solution, cells were stained with crystal violet to reveal plaques. Plaques were counted and the viral titer was represented as plaque-forming units (PFU)/mL. 

## 3. Results

### 3.1. Time-Course of NiV-Induced Syncytia Formation

During the replication of enveloped viruses, viral attachment and fusion proteins, together with receptors expressed on neighboring cell membranes, can lead to syncytia formation [19]; this is a hallmark of NiV infection, where syncytia can be observed in vitro but also in vivo in multiple organs such as the lungs, the brain, or the kidneys, amongst others [20]. Hence, the fusion of neighboring cells can generate multinucleated giant cells during NiV infection that allow the direct cell-to-cell transmission of NiV particles without the necessity for budding, thus accelerating the dissemination of the virus [21]. Thus, syncytia formation during NiV infection can be used to determine the progression of infection and virus spread.

**Figure 2:**
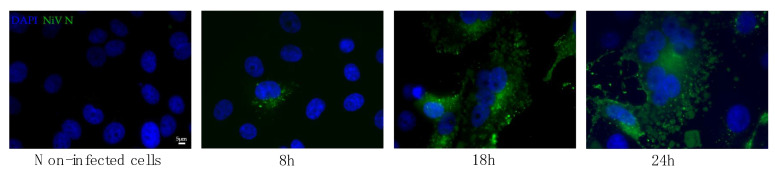
Time-course of syncytia formation during NiV infection. Vero E6 cells were infected at an MOI of 0.01 and fixed at 8, 18 and 24 h p.i. Non-infected cells were also fixed at 24 h as a negative control. Cells were then stained with 5F12 anti-NiV N antibody (green) and DAPI stain (blue) in order to visualize the syncytia formation. Scale bar 5 µm.

Since the formation of syncytia can rapidly destroy the cellular monolayer, we performed time-course experiments in order to determine the best time point at which to fix cells for NiV N antigen detection by immunofluorescence. As shown in Figure 2, while infection with NiV was detected as early as 8 h p.i., 24 h was the best time point for detection since it represented infection without excessive damage to the cells, thus allowing IF visualization. At 48 h p.i., almost no cells were left on the infected slides; therefore, these data are not shown. 

### 3.2. Validation of the Inactivation Methods

**Figure 3:**
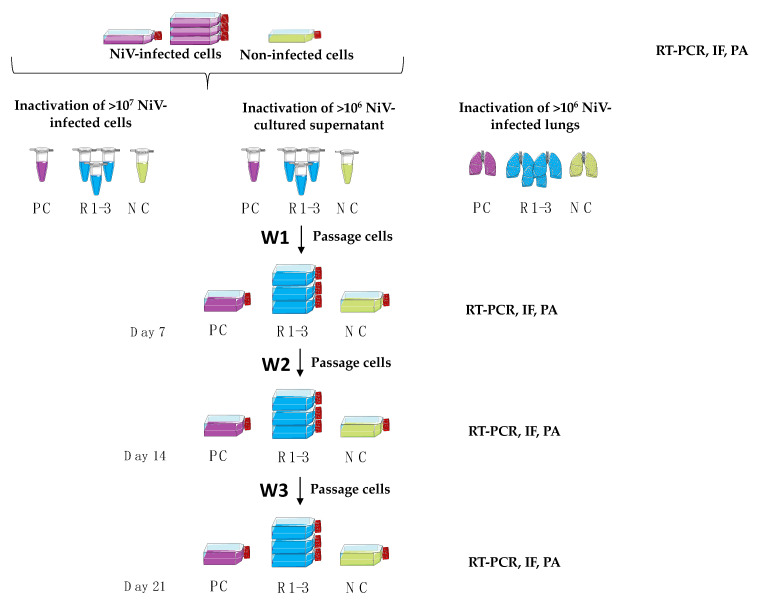
Schematic representation of the validation process of NiV inactivation techniques. Triplicates (R1, R2 and R3) of either >1 × 10^7^ PFU/mL NiV-infected Vero E6 cells, supernatant with >1 × 10^6^ PFU/mL NiV, or NiV-infected mouse lungs were inactivated following the respective protocols. An uninfected negative control (NC, uninfected cells, organs, or medium) was equally treated, while an additional specimen of each method was not inactivated to serve as a positive control (PC). To remove cytotoxic agents after inactivation, non-lysed cells were washed with PBS, while lysed cells and supernatant samples were diluted in PBS and concentrated to a final volume of 1 mL using Amicon centrifugal filters. Thereafter, cleared cells or supernatant were used to inoculate fresh Vero E6 cells for a week. Each sample was passaged weekly over a three-week period, transferring cells and supernatant of each sample to a new cell culture flask. During this period, harvested supernatants were screened by RT-qPCR, plaque assay (PA), and immunofluorescence microscopy (IF). Baseline samples (day 0) of supernatant and cells prior to inactivation were collected and analyzed to ensure initial infection. Figure created with smart.servier.com.

Prior to inactivation, virus titers were quantified by a plaque assay and the presence of NiV RNA and its infectious capacity were confirmed by RT-qPCR and a plaque assay, respectively, for each sample set (Figure S1). A total of nineteen inactivation conditions, summarized in Table 1, were tested on cells, supernatants or organs (lungs), and samples were analyzed for the presence of infectious NiV over 3 weeks (Figure 3).

In order to detect NiV persistence in Vero E6 cells after the inactivation process, besides the evaluation of CPE, we performed molecular diagnostics of NiV by RT-qPCR, with one immunological diagnostic technique to detect NiV N protein by IF and one functional assay to assess the potential infectious capacity of the inactivated samples by PA.

**Figure 4:**
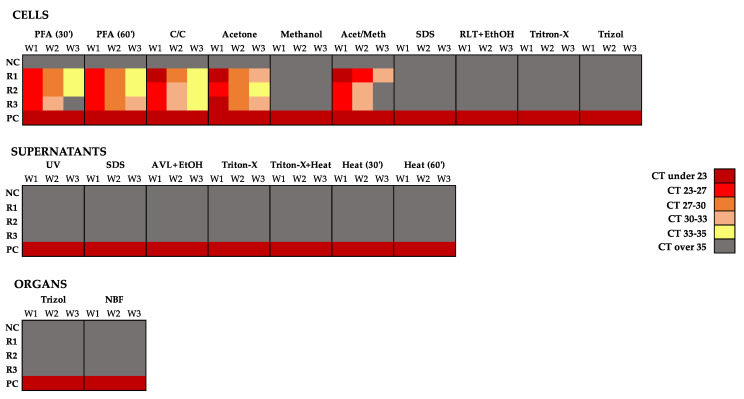
NiV quantification by RT-qPCR. NiV genomes contained in the samples collected at week 1 (W1), week 2 (W2) and week 3 (W3) were quantified by RT-qPCR targeting the NiV N gene. The cycle threshold (CT) was semi-quantified based on standard curves and represented as a heat map. Triplicates (R1, R2 and R3), uninfected negative control (NC) and positive control (PC).

There are various methods that are suitable to preserve genetic material while inactivating the potential infectious capacity of the pathogen. In our study, we used inactivation methods that involved: two organic solvents (acetone and methanol) and a mixture of both, fluoropolymers/aldehyde fixatives (PFA, C/C and NBF), detergents (SDS and Triton-X 100) and guanidinium isothiocyanate-containing reagents (AVL, RLT and Trizol). As shown in Figure 4, the inactivation of NiV-infected cells with PFA, C/C and all the inactivation methods involving organic solvents, except for methanol inactivation, presented detectable NiV RNA genome levels that subsequently decreased over the 21-day propagation period (three passages). In contrast, when applying other inactivation methods such as SDS, RLT + EtOH, Triton-X 100 or Trizol reagent, no detectable NiV RNA genome was observed.

It has previously been shown that acetone better preserves both DNA and RNA compared to EtOH, where the extent of degradation of the genomic material is higher [22]. These differences in preservation, which are even more accentuated when the organic solvent is diluted in other liquids, correlate with the differences found in using acetone or methanol to inactivate NiV-infected cells. While acetone preserved the NiV genome to a certain extent, cells treated with methanol were not shown to contain NiV genome traces when genome quantification was performed by RT-qPCR analysis. As expected, the combination of both solvents led to an intermediate degree of NiV genome preservation.

The fact that we observed a decrease in NiV genome levels over the three passages was an expected result, since the infected fixed cells contained the fixed virus. However, the virus was inactivated and therefore couldn’t replicate, and it (the genome) was thus diluted when cells were passaged over 3 weeks.

Supernatants and organ samples did not show any presence of NiV genome in either the negative control or any of the triplicate samples, independently of the method chosen. Importantly, in all cases, the positive control presented high levels of NiV genome over the three-week period of propagation. 

**Figure 5:**
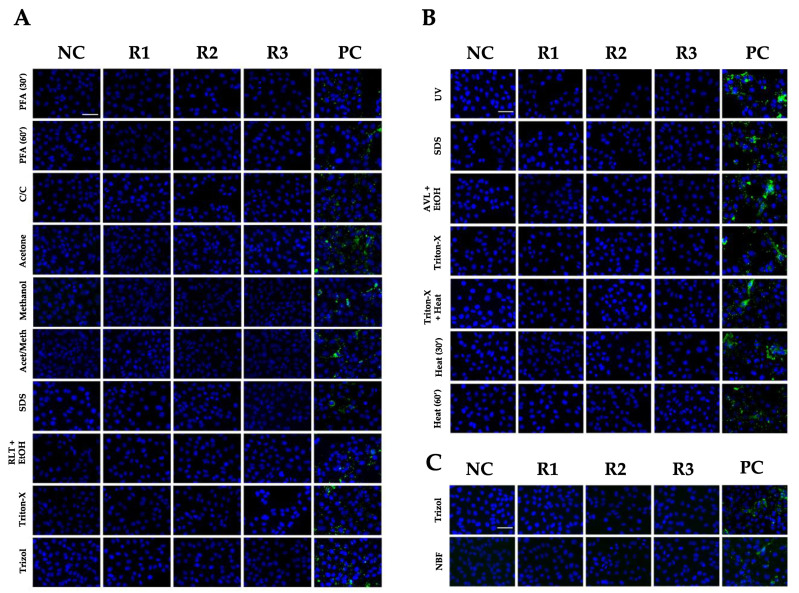
Immunofluorescence analysis of NiV N expression in Vero E6 cells. Media collected from inactivated NiV-infected cells (**A**), supernatants (**B**) and organs (**C**) were used to treat Vero E6 cells for 24 h. Images correspond to treatment of Vero E6 cells the last week of propagation (W3). Fixed Vero E6 cells were stained by indirect immunofluorescence using 5F12 anti-NiV N antibody (green) and DAPI stain (blue). Triplicates (R1, R2 and R3), uninfected negative control (NC) and positive control (PC). Scale bar: 50 µm.

To assess viral replication in these cultures, aliquots of supernatant for each condition were harvested weekly and used to inoculate fresh Vero E6 cultures. At 24 h p.i., the media were removed and the cells were fixed and stained for the presence of NiV N protein by indirect immunofluorescence. As shown in Figure 5, after three weeks of propagation, the negative control and the culture supernatants of inactivation methods applied on cells, supernatants, or organs did not show any presence of NiV N protein by IF and, thus, no viral replication was detected at week 3 of propagation. In contrast, NiV nucleoprotein was present in the control samples that were not subjected to inactivating treatments (positive control). Differences in intensity may be due to NiV CPE, which may damage the cells, thus preventing observation of the full extent of the infection in some cases. This indicates that all the inactivation methods performed in cells, supernatants and organs successfully inactivated NiV.

**Figure 6:**
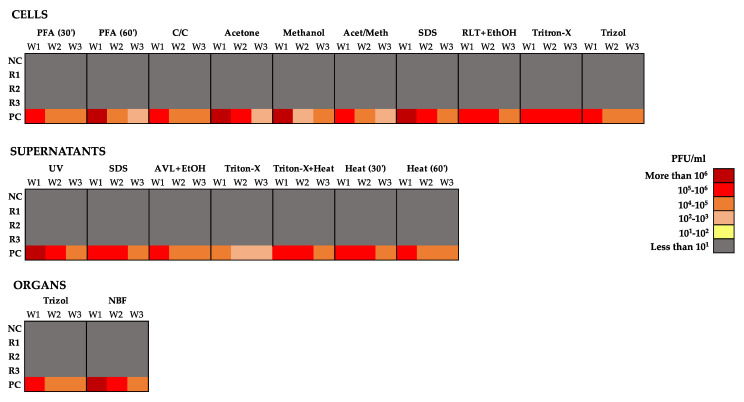
NiV replication capacity over three passages after inactivation. NiV infectious capacity of the samples collected at week 1 (W1), week 2 (W2) and week 3 (W3) was quantified by plaque assay (PA), and expressed in plaque formation units (PFU) per ml in a heat map. Triplicates (R1, R2 and R3), uninfected negative control (NC), positive control (PC).

To further determine viral titers, we performed plaque assays with the weekly collected supernatants of the inactivation methods tested. In all nineteen inactivation conditions tested, all replicates (R1-3) for all tested samples and their respective negative control did not show evidence of infection by PA (Figure 6). In contrast, NiV was detected in all positive controls of all inactivating conditions. The decrease in titers detected in the positive controls over the 3 weeks is most likely due to the fact that during the propagation phase, supernatants were collected at day 7 post-infection rather than at day 3, which is the optimal point to do so. Moreover, no cytopathic effect was observed during the incubation of the inoculum over the 3 weeks; in contrast, all flasks containing the positive control had a disrupted cell monolayer. 

## 4. Discussion

During diagnostics and research in BSL-4 facilities, it is necessary to have safe and well-established inactivation procedures to enable further downstream analyses of samples outside of these high-containment laboratories. However, current published validated methods for inactivating henipaviruses are very limited. As observed previously with inactivation procedures, there are many factors that contribute to efficient and safe inactivation, namely, the specific pathogen being inactivated, the contact time between the inactivating reagent and the pathogen, the type of sample being inactivated, the specific inactivation reagent, the viral load, the protein content of the sample, the concentration of the inactivation product, the mixing and homogenization efficiency of the inactivating reagent, and the reproducibility of the procedure. For this reason, we validated the most commonly used inactivation techniques in different sample types (cells, supernatants and organs).

In general, the validation of inactivation methods is challenging due to the presence of chemicals that not only destabilize the virus in the sample, but also damage the cells in culture upon which the sample is inoculated when testing for any residual infectious virus particles following inactivation. It is possible to decrease or remove the concentration of cytotoxic reagents by dialysis, for instance; however, the incubation time of this process can alter the viral titer, thus distorting the real virus titer and the real efficacy of the inactivation method. Moreover, some chemicals are difficult to remove with this technique [23,24]. In light of this difficulty, we used a recently described in vitro protocol where the use of Amicon^®^Ultra-15 100K Centrifugal Filter Devices allows both virus sample concentration and the simultaneous removal of cytotoxic substances via ultrafiltration [15]. Importantly, this method ensured a high virus recovery rate in a non-toxic, small sample volume that could easily be added to fresh cells in order to test the remaining infectivity of the samples.

In addition, some other inactivating agents, once thought to be efficient, have previously been shown to be unsuccessful at inactivating all virus particles. This is the case for Ebola virus (EBOV) where it was shown that AVL solution alone was not able to fully inactivate virus-containing samples, and that AVL needed to be combined with EtOH in order to achieve the complete inactivation of EBOV [25]. This same study also showed that neither a 1:4 ratio of solution:ethanol nor heat inactivation led to viral inactivation.

Considering that viral inactivation is often essential for the downstream analysis or processing of samples, it is important to be able to assess the degree to which viral infectivity is eliminated and that viral transmission can no longer occur. Moreover, it is also important to take into account that there is no infallible procedure for viral detection, since all viral screening methods are unable to detect virus particles or infection below a certain level due to the intrinsic detection limits of each technique. In this sense, in this study, several methods including RT-qPCR, IF and PA were employed to ensure the maximum sensitivity of NiV detection.

In order to ensure a total loss of the infectiousness of a sample whilst still preserving antigenic and/or genomic material, different methods were tested in distinct sample types. It is important to distinguish between virus removal and virus inactivation. While genetic material or viral proteins may still be detected after inactivation, this does not imply that they are infectious. Fourteen of the methods tested here showed no remains of either viral protein or genetic material at levels detectable with the techniques used, whereas five inactivation methods showed the presence of inactivated genomic material that decreased over time due to degradation or dilution during cell passaging. However, these methods have proven their virucidal activity, since the samples were effectively inactivated and did not present any infectious capacity, as assessed through IF or PA.

All of the procedures tested in this study were destined to effectively remove the infectivity of the samples and, in order to ensure the safety of these procedures, samples were maintained over 3 weeks. During this time, inactivated sample cultures were left in contact with freshly split Vero cells for 7 days before passaging again. This ensured that any potential remaining non-inactivated virus particles could easily be amplified. With regard to NiV, this is a rather long time period given that the NiV life cycle lasts approximatively 8 h [26]; however, for other viruses, replication is slower, e.g., the EBOV replication cycle takes around 30 h [27]. One of the limitations that we encountered was that the high cytopathic effect of NiV rendered the validation of the methods difficult. At 48 h p.i. with NiV, the cell monolayer was damaged with most of the cells destroyed and the remaining cells formed large syncytia. We therefore had to limit the incubation period of our potentially inactivated samples to 24 h, which was shown to still be more than sufficient to observe the infectious capacity of the samples by IF. For titration, samples were left with the methylcellulose overlay for 3 days, as previously described [28].

With our study, we have established fourteen inactivation methods for NiV that have been applied to distinct specimen types (cells, supernatants and organs), thus targeting different research purposes. These procedures include physical (UV irradiation, heat inactivation), chemical (PFA, C/C reagent, acetone, methanol, acetone/methanol, SDS, RLT, AVL, Triton-X 100, Trizol and NBF) or combined (heat and SDS combined) methods in nineteen different conditions. We tested the conditions that are most commonly used during experimentation to further process samples outside BSL-4 containment by flow cytometry, RT-qPCR, Western blot and ELISA, amongst others. The relevance of this study lies in the fact that it considerably increases the information available on NiV inactivation methods validated by BSL-4 facilities, which may be used as a reference by other laboratories not only for the inactivation of related viruses, such as Hendra virus or for structurally similar lower containment paramyxoviruses, but also potentially for other pathogens. However, further validation of other inactivation methods should be performed in the future. For instance, the inactivation of NiV contained in sera or in sucrose solutions should be validated since it has previously been shown that NiV is more persistent when contained in artificial palm sap due to the high sugar content of this liquid [29]. Moreover, we tested our samples at a low concentration of sera (5%); therefore, media or solutions containing higher serum concentrations should also be tested, since it has previously been shown that serum can affect the inactivation capacity of certain reagents such as SDS during EBOV inactivation [30]. While, in this study, NiV-infected samples were tested, the information provided concerning the inactivation protocols and their efficacy may also be relevant for laboratory personnel or health workers for the inactivation treatment of other samples/surfaces including fomites, virus-exposed surfaces or laboratory equipment, amongst others, particularly for lower containment paramyxoviruses or clinical samples. Any such protocol would obviously still require testing and validation in line with the requirements of specific laboratory/institute or country guidelines. Lastly, it is important to consider that inactivation reagents are subject to natural variations, which is why it is vital to use inactivation reagents that have been well conserved, properly stored, and that have not expired; otherwise, the inactivation procedure can be inefficient and lead to inaccuracies. This also applies to UV light where time shortens the lamp’s lifespan, thus failing to ensure proper decontamination.

We envisage that the wide range of methods tested in our study will help to improve or approve Standard Operating Procedures (SOPs) for the inactivation of other viruses, especially negative, single-stranded enveloped viruses, thus allowing further processing after virus inactivation. 

## Figures and Tables

**Table 1:** Inactivation methods tested in NiV-infected samples.

Inactivated Methods	Sample Yype	Inactivation Conditions
PFA	Cells	30 min, RT
PFA	Cells	60 min, RT
Cytofix/Cytoperm (C/C)	Cells	30 min, RT
Acetone	Cells	30 min, RT
Methanol	Cells	30 min, RT
Acetone/Methanol	Cells	30 min, RT
SDS	Cells	10 min, 95 °C
RLT + EtOH	Cells	a.m.i.
Triton-X 100	Cells	20 min, RT
Trizol	Cells	20 min, RT
UV light	Supernatant	1 h, RT
SDS	Supernatant	10 min, 95 °C
AVL + EtOH	Supernatant	a.m.i.
Triton-X 100	Supernatant	20 min, RT
Triton-X 100 + Heat	Supernatant	30 min, 56 °C
Heat	Supernatant	30 min, 56 °C
Heat	Supernatant	60 min, 56 °C
Trizol	Organ	20 min, RT
NBF	Organ	2 × 24 h + 48 h PBS, RT

RT—room temperature; a.m.i.—according to manufacturer’s instructions.

## Data Availability

Not applicable.

## Supplementary Materials

The following supporting information can be downloaded at: https://www.mdpi.com/article/10.3390/v14051052/s1, Figure S1: NiV replication capacity before inactivation.
