# Supplementary material for: Inactivation Methods for Experimental Nipah Virus Infection
Source: Viruses. 2022 May 15;14(5):1052. doi: 10.3390/v14051052 (PMC9145063; doi:10.3390/v14051052)
Supplement: Supplementary file 1 [file viruses-14-01052-s001.zip › viruses-1656559-supplementary.pdf]

Supplementary Figures

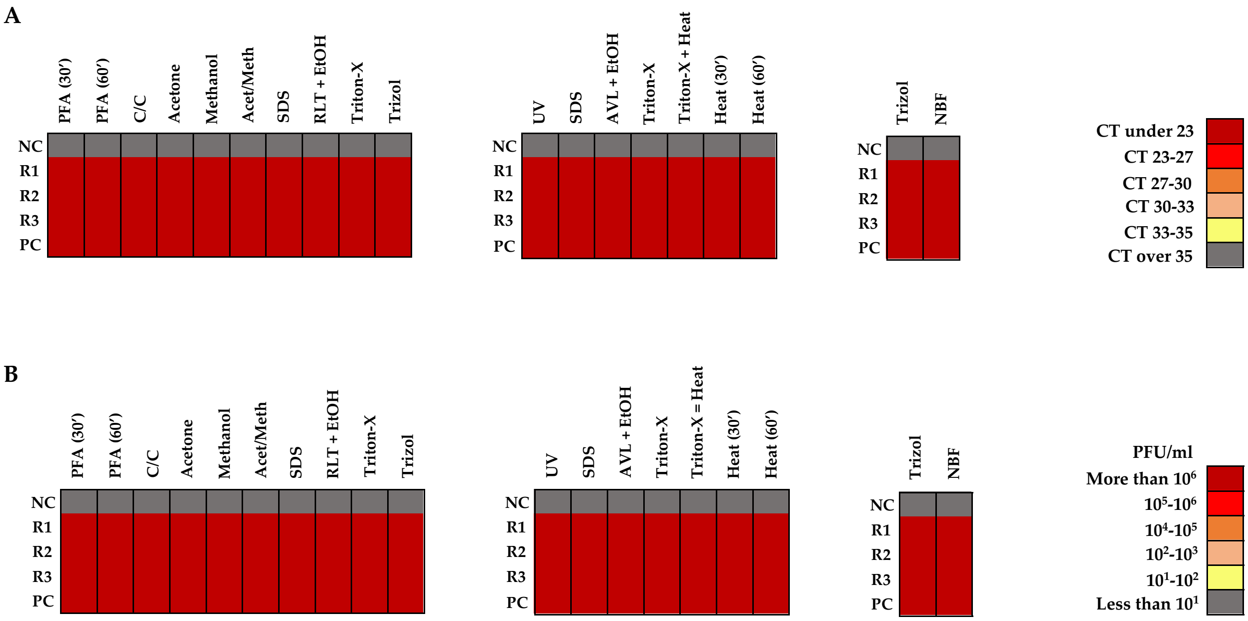

Figure S1. NiV replication capacity before inactivation. NiV infectious capacity of the samples collected before inactivation was quantified by RT-PCR (A) targeting the NiV N gene. The cycle threshold (CT) was semi-quantified based on standard curves and represented as a heat-map. (B) A plaque assay was used in order to quantify the infectious particles contained in the samples before inactivation. The plaques were counted and expressed in plaque formation units (PFU) per ml in a heat map. Triplicates (R1, R2 and R3), uninfected negative control (NC) and positive control (PC).
